# Differential expression of Oct-4, CD44, and E-cadherin in eutopic and ectopic endometrium in ovarian endometriomas and their correlations with clinicopathological variables

**DOI:** 10.1186/s12958-020-00673-1

**Published:** 2020-11-20

**Authors:** Ceyda Sancakli Usta, Gulay Turan, Cagla Bahar Bulbul, Akin Usta, Ertan Adali

**Affiliations:** 1 Department of Obstetrics and Gynecology, School of Medicine, Balikesir Univesity, Cagis Yerleskesi, Bigadic yolu 17. km, 10145 Balikesir, Turkey; 2 Department of Pathology, School of Medicine, Balikesir Univesity, Balıkesir, Turkey

**Keywords:** Endometriosis, Oct-4, CD44, E-cadherin, Endometrial tissue

## Abstract

**Background:**

Endometriosis is an estrogen-dependent inflammatory disease that often causes infertility and chronic pelvic pain. Although endometriosis is known as a benign disease, it has demonstrated characteristics of malignant neoplasms, including neoangiogenesis, tissue invasion, and cell implantation to distant organs. Octamer-binding protein 4 (Oct-4) is a molecular marker for stem cells that plays an essential role in maintaining pluripotency and self–renewal processes in various types of benign and malignant tissues. CD44 is a multifunctional cell surface adhesion molecule that acts as an integral cell membrane protein and plays a role in cell–cell and cell–matrix interactions. E-cadherin is an epithelial cell–cell adhesion molecule that plays important role in the modulation of cell polarization, cell migration, and cancer metastasis. The aim of this study was to investigate the expression patterns of Oct-4, CD44, and E-cadherin in eutopic and ectopic endometrial tissues from women with endometrioma compared to control endometrial tissues from women without endometrioma.

**Methods:**

In the present study, Oct-4, CD44, and E-cadherin expressions were evaluated in eutopic and ectopic endometrial tissue samples from women with endometrioma (*n* = 32) and compared with those of control endometrial tissue samples from women without endometrioma (*n* = 30).

**Results:**

Immunohistochemical expression of Oct-4 was significantly higher in the ectopic endometrial tissue samples of women with endometrioma than in the control endometrial tissue samples (*p* = 0.0002). Conversely, CD44 and E-cadherin expressions were significantly lower in the ectopic endometrial tissue samples of women with endometrioma than in the control endometrial tissue samples (*p* = 0.0137 and *p* = 0.0060, respectively). Correlation analysis demonstrated significant correlations between Oct-4 expression and endometrioma cyst diameter (*p* = 0.0162), rASRM stage (*p* = 0.0343), and total rASRM score (*p* = 0.0223). Moreover, CD44 expression was negatively correlated with the presence of peritoneal endometriotic lesions (*p* = 0.0304) while E-cadherin expression was negatively correlated with the presence of deep infiltrating endometriosis (*p* = 0.0445).

**Conclusions:**

Increased expression of Oct-4 and decreased expression of adhesion molecules in endometriotic tissues may contribute to the development and progression of endometriosis.

## Background

Endometriosis is one of the most common chronic inflammatory diseases in reproductive–age women. Its prevalence is 5–10% and can cause chronic pelvic pain and infertility [1]. Ovarian endometrioma is the most prevalent subtype of endometriosis. Endometriosis is currently treated medically, surgically or with a combination of both medical and surgical techniques.

Although it is highly prevalent and decreases quality of life, the molecular and cellular mechanisms of endometriosis are still unknown. However, recent studies have shown that endometrial stem cells play a role in the etiopathogenesis of endometriosis [1–4].

The human endometrium is a dynamic tissue with a high regenerative capacity [5]. Previous studies have suggested that this high regenerative capacity is mediated by endometrial stem cells [6]. Octamer-binding protein 4 (Oct-4) is a molecular marker for stem cells that plays an essential role in maintaining cell pluripotency and self–renewal processes in various types of benign and malignant tissues [2–4].. Previous studies have demonstrated that Oct-4 is expressed in many types of pluripotent cells, such as embryonic stem cells, germ cells, and endometrial cells [1, 7, 8].

Endometriosis is known as a benign condition. However, it has demonstrated characteristics of malignant neoplasms, including neoangiogenesis, tissue invasion, and cell implantation to distant organs [9]. Retrograde menstruation is the most widely hypothesized cause for the implantation of endometrial tissue in the peritoneal cavity and the survival of endometrial cells [10]. Recent studies have suggested that the loss or reduced expression of adhesion proteins, including CD44 and E-cadherin, is associated with benign and/or malignant cell proliferation, cell migration, tumor invasion, and cancer metastasis in a wide array of cells and tissues [11, 12]. CD44 is an integrated membrane protein that acts as a multifunctional cell surface adhesion molecule and plays a role in cell–cell and cell–matrix interactions [13, 14]. Previous studies have shown thatin women with endometrioma, the expression of CD44 is lower in ectopic endometrial tissues than in eutopic endometrial tissues [12]. E-cadherin is an adhesion glycoprotein that is expressed by eutopic and ectopic endometrium [15]. It is an epithelial cell–cell adhesion molecule that modulates a wide variety of processes, including cell polarization, cell migration, and cancer metastasis [16, 17]. Decreased expression of E-cadherin, which could promote malignant transformation, tumor invasion, and metastasis, has been observed in various cancers [18].

Few studies have investigated adhesion molecule expression in eutopic and ectopic endometrial tissues and those that have produced varied results. Therefore, we investigated the expression patterns of Oct-4, CD44, and E-cadherin in eutopic and ectopic endometrial tissues from women with endometrioma, as well asthe detailed correlations of these patterns with clinicopathological findings.

## Methods

### Patients

In this cross-sectional study, all subjects gave informed consent before participation. Eutopic (endometrial cells) and ectopic endometrial tissue samples (endometrioma cyst wall tissue) were obtained of 32 women with endometrioma during laparoscopic surgery. Control endometrial tissue samples were obtained from uterine cavity of 30 women who were undergoing laparoscopic surgery for benign indication including myoma uteri, tubal sterilization, and serous and mucinous cystadenoma of the ovary. This study was conducted in Training and Research Hospital of Balikesir University. The investigation protocol was approved by the institutional Ethical Committee of the Balikesir University, School of Medicine before starting the study. The study protocols were in accordance with the Helsinki Committee requirements. Patients who had malign diseases, pelvic infections, chronic systemic diseases, advanced pelvic and abdominal adhesion, pelvic radiation exposure, and hormone replacement therapy before 3 month the surgery were excluded from the study.

### Tissue specimens preparation and examination

All tissue specimens of endometrioma and control groups were fixed in formalin and subsequently embedded in paraffin. All paraffin-embedded tissue blocks were then cut into 4-μm thick sections and stained with hematoxylin and eosin after the deparaffinization.

### Immunohistochemistry

Immunohistochemical (IHC) staining for Oct-4, CD44 and E-cadherin were performed on eutopic endometrium, ectopic endometrium and control endometrial tissue samples. All tissue preparation and IHC procedure were performed as described previously [19]. The primary antibodies against Oct-4 (dilution 1:100), CD44 (dilution 1:50) and E-cadherin (dilution 1:200) (Dako, Glostrup, Denmark), chromogen aminoethylcarbazole substrate kit (AEC kit; Zymed Laboratories), mounting medium solution (Zymed 00–8030) and super vision assay kit (SV0002–1, Boster, Pleasanton, CA, USA) were used in this study.

### Evaluation of all tissue samples

The all tissue samples underwent gross and histologic examinations by an experienced histopathologist (G.T.). Gross examinations were performed on the basis of endometrioma size, bilaterality, and the presence of deep infiltrating endometriosis lesions. Histologic examinations were performed on the basis of staining characteristics of primary antibody and cyclic phases of eutopic and control endometrial tissues that were evaluated in accordance with Noyes’ Criteria [20]. During the evaluation, histopathologis was blinded to the clinical variables.

During the histological examination, 10 slide fields were selected randomly and analyzed using a biomicroscope (Olympus BX48, Tokyo, Japan) with an image analysis system (Nis Elements Advantages Research Microscope Imaging Software, Nikon Enstruments Europe BV, Amsterdam, Netherlands). Brown-color staining intensity revealed the presence of Oct-4, CD44 and E-cadherin expressions. In all patients with endometrioma, the eutopic and ectopic endometrial tissue samples showed strong immunoreactivity for Oct-4, CD44 and E-cadherin. The used image analysis system consisted of a computer with hardware and software for image acquisition and analysis, a spot insight camera, and an optical microscope. The method requires preliminary software procedures involving spatial calibration (on a micron scale) and setting of color segmentation for quantitative color analysis.

### Quantitation of staining intensity

All tissue samples of each group were evaluated. In each high-power field, numbers of Oct-4 positive cells were counted. The numbers of immunoreactive cells in all 10 high-power fields were averaged to come up with a score for each sample. Cytoplasmic staining of CD44 was semiquantitatively scored as 0 (absent), 1+ (sporadic or weak reaction of single cells), 2+ (moderate and heterogeneous staining pattern with < 50% positive cells), or 3+ (strong staining of > 50% of cells) for a given cell type. Cell membrane staining of glandular cell for E-cadherin, the staining intensity of the glandular epithelial cells was estimated as follows: 0: negative; 1: weak staining; 2: moderate staining; and 3: strong staining. The positivity ratio was evaluated; the number of pixels reflects the expression level of the detected antigen and can also be expressed as percentage of the entire image pixel amount. < 80 pixels reflects: weak (1+), 80–200 pixels reflects: moderate (2+) or > 200 pixels reflects: strong (3+).

For statistical analysis, comparisons of metric or categorical variables between studied group and controls were done by indepentent t test, Mann-Whitney test or Fisher exact test. Qualitative data are expressed in percentage (%) and quantitative data are expressed as the means ± Standard deviation (SD). The correlation analysis were performed with spearmans’ rank correlation test. *p* values of 0.05 were considered to be statistically significant. MedCalc Statistical Software Programme Version 19.0.4 (Ostend, Belgium) was used.

## Results

In this cross-sectional study, histopathological specimens of eutopic and ectopic endometrial tissues from 32 women with endometrioma were evaluated and compared with control endometrial tissues from 30 women without endometrioma. The mean age of participants was 29.9 ± 3.6 years in the endometrioma group and 31.8 ± 4.7 years in the control group. There were no significant differences in age (*p* = 0.0841) or parity (*p* = 0.5140) between the two groups. Participants’ characteristics are summarized in Table 1.

**Table 1:** Demographic characteristics of patients in the groups

Variables	Endometriosis (***n***=32)	Control (***n***=30)	***P*** value
Median age, year (mean)	29.9±3.6	31.8±4.7	0.0841^a^
Parity, n (%)			
Nulliparity	21 (65.6)	22 (73.3)	0.5140^b^
Multiparity	11 (34.4)	8 (26.7)	
Previous abdominal surgery, n (%)			
Yes	5 (15.6)	3 (10.0)	0.5125^b^
No	27 (84.4)	27 (90.0)	
Median level of CA125 (U/mL), median (min-max)	81.5(44.1-218)	33.7 (12-66.3)	<0.001^a^
Menstruel cycle phases, n			
Early proliferative	7	6	0.9922^b^
Late proliferative	6	6	
Early secretory	5	6	
Mid secretory	8	7	
Late secretory	6	5	
Median endometrioma cyst diameter, cm (min-max)	56.3 ±12.2	-	
Bilaterality of endometrioma, n (%)			
Yes	9 (28.1)	-	
No	23 (71.9)		
r ASRM stage, n (%)			
III	24 (75.0)	-	
IV	8 (25.0)		
r ASRM total score (mean±SD)	35.3±11.8	-	
Cul-de-sac obliteration, n (%)			
No	15 (46.9)	-	
Partial	12 (37.5)		
Complete	5 (15.6)		
Presence of Peritoneal Endometriotic Lesions, n (%)			
Yes	9 (28.1)		
No	23 (71.9)		
Presence of Deep Infiltrating Endometriosis			
Yes	4 (12.5)		
No	28 (87.5)		

^a^Mann-Whitney test

^b^Chi-squared test

**Fig. 1:**
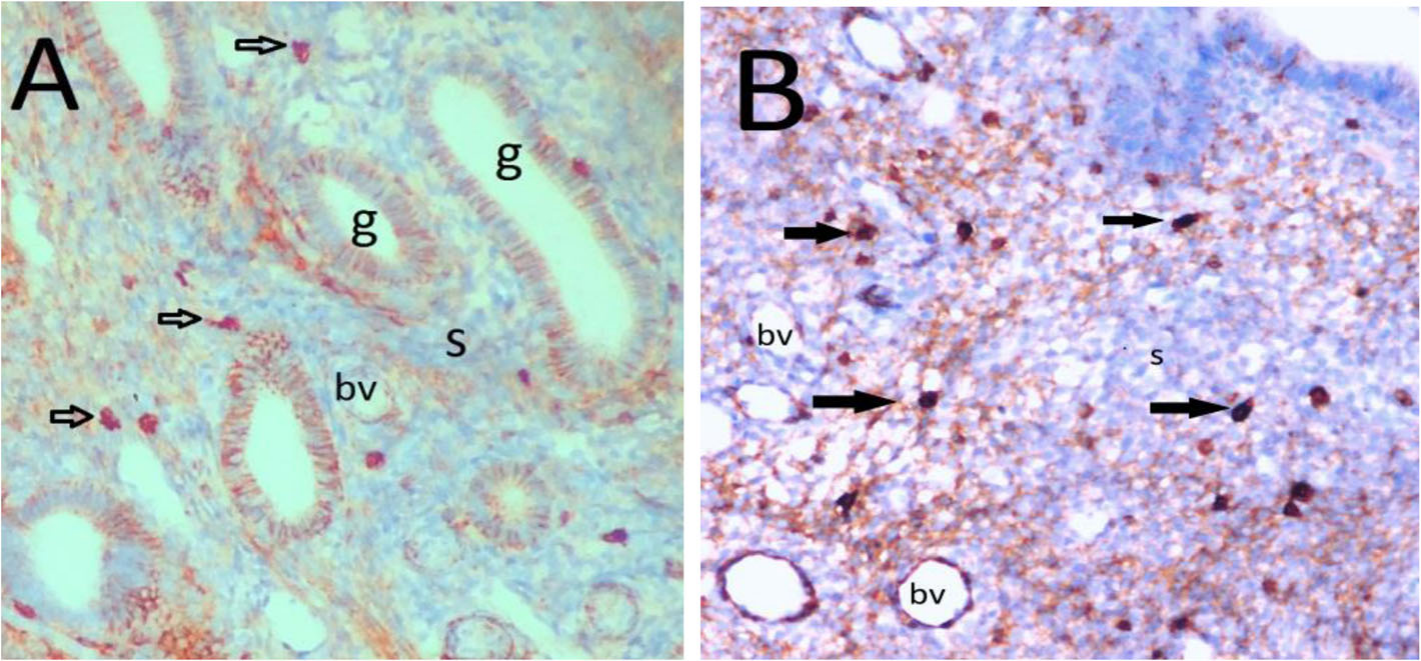
Different expression of Oct-4 in endometrial tissues by immunohistochemistry (× 200). **a** Weak expression of Oct-4 in control endometrial tissue from woman without endometrioma (light brown staining, score 1). **b** Strong expression of Oct-4 in ectopic endometrial tissue from woman with endometrioma (dark brown, score 3). g, endometrial gland; s, endometrial stroma; bv, blood vessel, black arrow Oct-4 positive cells

**Fig. 2:**
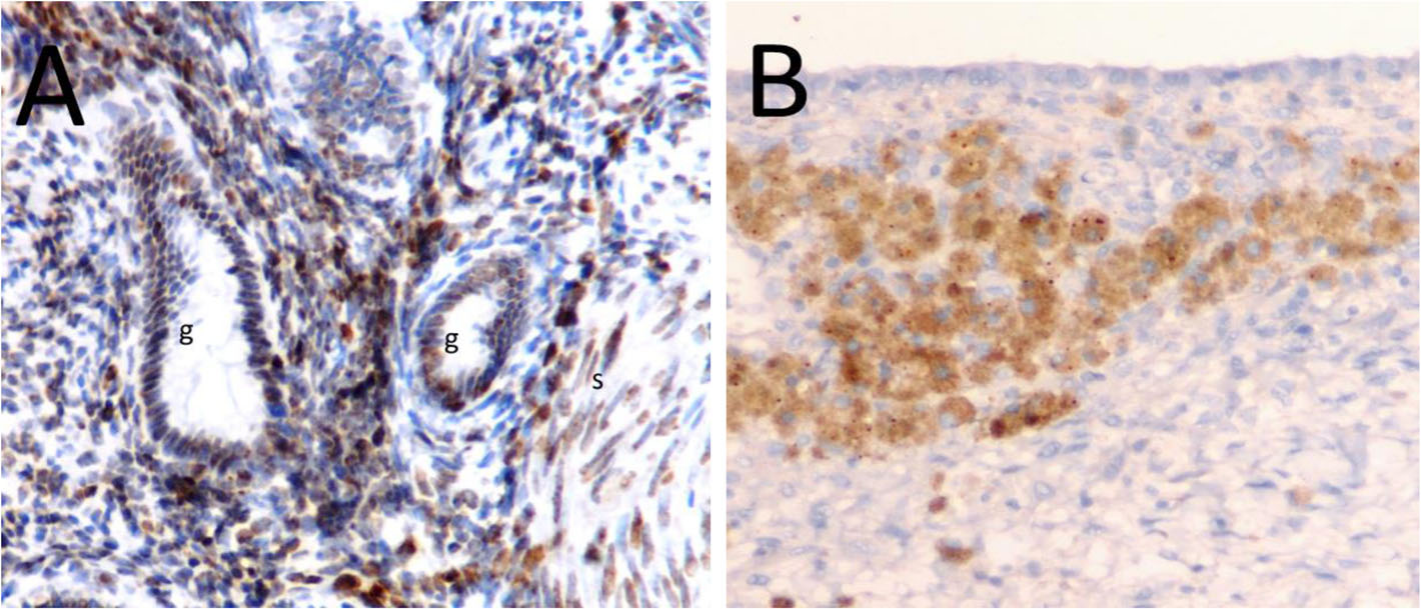
CD44 expression in eutopic and ectopic endometrial tissue by immunohistochemistry (× 200). **a** CD44 expression in eutopic endometrial tissue from woman without endometrioma revealing strong staining in columnar epithelium, and stroma (dark brown, score 3). **b** CD44 expression in ectopic endometrial tissue from woman with endometrioma revealing staining in some columnar cells and stromal cells (light brown, score 1). g, endometrial gland; s, endometrial strom

**Fig. 3:**
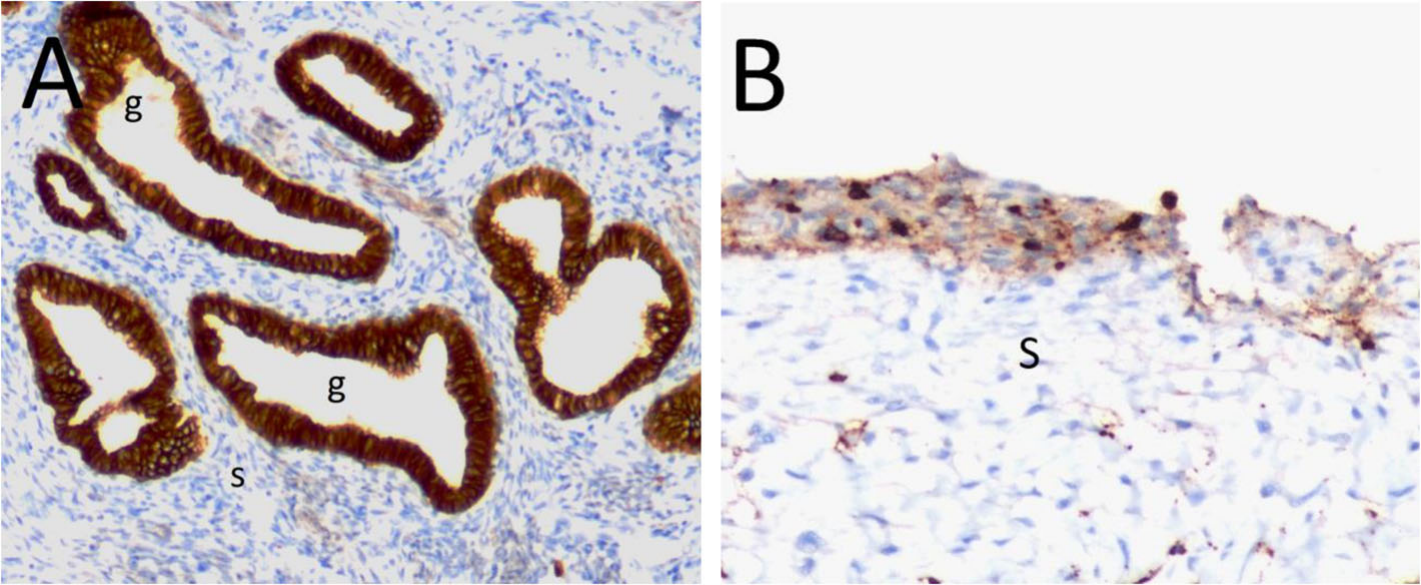
E-cadherin expression in eutopic and ectopic endometrial tissue by immunohistochemistry (× 200). **a** E-cadherin expression in eutopic endometrial tissue from woman without endometrioma revealing strong staining in glandular epithelium (dark brown, score 3). **b** E-cadherin expression in ectopic endometrial tissue from woman with endometrioma revealing staining in some columnar cells (light brown, score 1). g, endometrial gland; s, endometrial stroma

In the immunohistochemical analysis of the eutopic and ectopic endometrial tissues, Oct-4 expression was predominantly localized in the nuclei of glandular and stromal cells (Fig. 1a and b). In contrast, CD44 expression was predominantly localized in the cytoplasm of glandular and stromal cells (Fig. 2a and b), and E-cadherin expression was predominantly localized in the cell membranes of glandular epithelial cells (Fig. 3a and b). The stromal cells of the eutopic and ectopic endometrial tissues were not stained for E-cadherin.

Notably, we found that women with endometrioma had significantly higher Oct-4 expression in their ectopic endometrial tissues than the control group (*p* = 0.0002). However, CD44 and E-cadherin expressions were significantly lower in the ectopic endometrial tissues of women with endometrioma than in the control group (*p* = 0.0137 and *p* = 0.0060, respectively). There were no statistically significant differences between the eutopic and control endometrial tissue samples. These results are shown in Table 2.

**Table 2:** Oct-4, CD44 and E- cadherin expressions in eutopic endometrial tissues of women with and without endometrioma.

Staining Score	Ectopic Endometrium (***n***=32)	Eutopic Endometrium (***n***=32)	Control Endometrium (***n***=30)	***P*** value*
**Oct-4**	5.67±2.44	3.12±1.89	3.50±1.75	**0.0002**
**CD44**	1.68±0.73	1.91±0,73	2.16±0.75	**0.0137**
**E-cadherin**	1.72±0.77	1.90±0,82	2.27±0.73	**0.0060**

*Mann-Whitney test

A correlation analysis of the Oct-4, CD44 and E-cadherin expressions and the participants’ clinicopathological variables revealed a significant correlation between Oct-4 expression and endometrioma cyst diameter (*p* = 0.0162), rASRM stage (*p* = 0.0343), and total rASRM score (*p* = 0.0223). Moreover, CD44 expression was negatively correlated with the presence of peritoneal endometriotic lesions (*p* = 0.0304), while E-cadherin expression was negatively correlated with the presence of deep infiltrating endometriosis (*p* = 0.0445;Table 3).

**Table 3:** Correlation analysis between Oct-4, CD 44 and E-cadherin expressions and clinicopathological variables of the patients with endometriosis

Variables	Oct-4	CD44	E-cadherin
Median age, year	0.2836 *p*=0.1157	0.2191 *p*=0.2283	0.0410 *p*=0.8235
CA125 level	0.0177 *p*=0.9234	0.1749 *p*=0.3382	-0.1431 *p*=0.4346
Endometrioma cyst diameter	**0,4219** ***P*=0.0162**	-0.1383 *p*=0.4503	-0.3361 *p*=0.0600
r ASRM stage	**0.3753** ***p*=0.0343**	-0.04969 *p*=0.7871	0.0237 *p*=0.8973
r ASRM total score	**0.4028** ***p*=0.0223**	-0.06161 *p*=0.7377	-0.2557 *p*=0.1579
Cul-de-sac obliteration	0.1052 *p*=0.5667	0.05185 *p*=0.7781	-0.2726 *p*=0.1312
Presence of Peritoneal Endometriotic Lesions	0.2277 *p*=0.2101	**-0.3833** ***p*=0.0304**	-0.2021 *p*=0.2673
Presence of Deep Infiltrating Endometriosis	0.08845 *p*=0.6302	-0.09759 *p*=0.5952	**-0.3576** ***p*=0.0445**
Oct-4	-	-0.1847 *p*=0.1507	**-0.3807** ***p*=0.0023**
CD44	-	-	**0.2626** ***p*=0.0392**

Spearman’s rank correlation

**Fig. 4:**
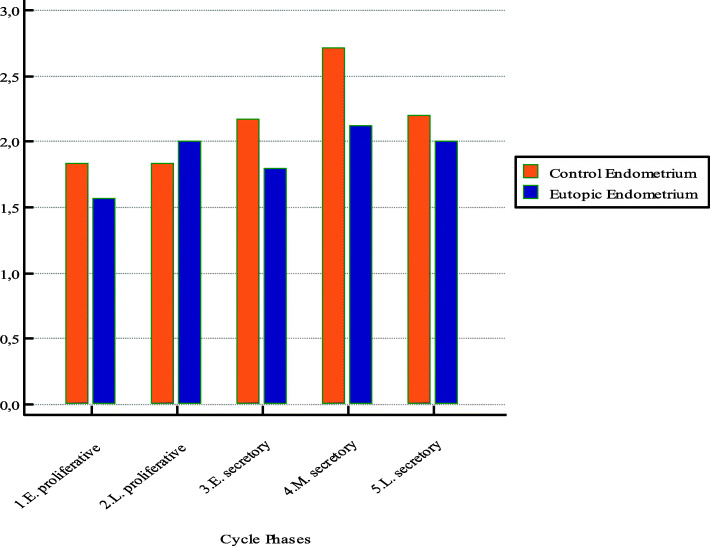
CD44 expressions in the different phases of the menstrual cycle in eutopic and control endometrial tissues. Bar graphs show a decreased expression of CD44 especially in early and mid-secretory phase of menstrual cycle in eutopic endometrial tissue of women with endometriosis

**Fig. 5:**
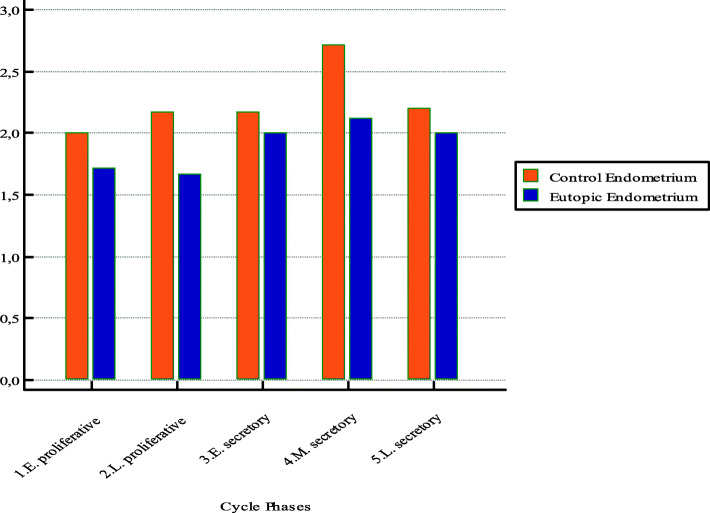
E-cadherine expressions in the different phases of the menstrual cycle in eutopic and control endometrial tissues. Bar graphs show a decreased expression of E-cadherin especially in mid-secretory phase of menstrual cycle in eutopic endometrial tissue of women with endometriosis

We also evaluated the cycle fluctuation of adhesion molecules in eutopic and control endometrial tissue, and we found that the expressions of CD44 and E-cadherin were lower in the proliferative phase of the menstrual cycle than in the secretory phase (Figs. 4 and 5). Additionally, CD44 and E-cadherin expressions were lower in the eutopic endometrial tissues than in the control endometrial tissues, especially in the secretory phase of the menstrual cycle. However, there were no significant differences in CD44 and E-cadherin expressions between the eutopic and control endometrial tissues (*p* = 0.0607 and *p* = 0.3291, respectively).

## Discussion

In the present study, we investigated the expression patterns of Oct-4, CD44, and E-cadherin in the eutopic and ectopic endometrial tissues of women with and without endometrioma and determined the correlations of these patterns with clinicopathological findings. According to the our results, Oct-4 expression was significantly higher in the ectopic endometrial tissues than in the eutopic and control endometrial tissues. In contrast, CD44 and E-cadherin expressions were significantly lower in the ectopic endometrial tissues than in the eutopic and control endometrial tissues. A correlation analysis showed that Oct-4 expression was correlated with endometrioma cyst diameter, rASRM stage, and total rASRM score. Additionally, CD44 expression was negatively correlated with the presence of peritoneal endometriotic lesions, and E-cadherin expression was negatively correlated with the presence of deep infiltrating endometriosis.

The molecular and cellular basis of endometriosis has long been an area of active investigation [21]. The most common hypotheses in the literature, for the etiopathogenesis of endometriosis include retrograde menstruation [22], coelomic metaplasia [23], embryonic rest, and lymphovascular metastasis [22]. However, none of these theories can explain the survival of endometrial cells found outside of the uterine cavity across all cases of endometriosis. It is most likely that a combination of several aberrant molecular and cellular processes and genetic predispositions cause implantation of the endometrial cells, invasion into the extrauterine tissue, and proliferation and survival of endometriotic lesions outside the uterine cavity [24].

The human endometrium is a dynamic tissue with a high regenerative capacity [5]. Previous studies have suggested that endometrial regeneration is mediated by endometrial stem cells [6]. Recent studies have also shown that eutopic and ectopic endometrial cells express several non-specific embryonic stem cell markers, including Oct-4, (sex determining region Y-box 2 (SOX-2), and c-KIT (CD117) [6, 8, 21]. Oct-4 is a transcription factor in the regulation of genes containing self-renewal and pluripotency capacities in embryonic stem cells and primordial germ cells [4, 25]. Oct-4 is also a key factor in the reprogramming of somatic cells to a pluripotent state [26]. Previous studies have demonstrated that overexpression of Oct-4 reflects benign development or malignant degeneration capacity in many tissues, including the prostate [27], and the endometrium [28]. Further, a recent study revealed relatively high expression of Oct-4 in the ectopic endometrium [28]. In a recent study conducted by Chang et al., the transcription of the Oct-4 gene was significantly up-regulated in the human ectopic endometrium, and Oct-4 positive pluripotent cells contributed to the pathological growth of ectopic endometrial tissue by stimulating the migration activity of endometrial cells [5]. Pacchiarotti et al. demonstrated that increased expression of Oct-4 in ectopic endometrial tissues may foster self-renewal, increased cell survival, and epithelial–mesenchymal transition in endometriosis [29]. In the present study, we found that Oct-4 expression was significantly higher in ectopic endometrial tissue than in eutopic or control endometrium. Moreover, we found that Oct-4 expression was positively correlated with endometrioma cyst diameter and rASRM stage. In light of aforementioned studies, our findings may indicate that overexpression of Oct-4 and its correlation with clinicopathological findings in endometriotic tissues may reflect tissue growth and neighborhood tissue destruction.

CD44 and E-cadherin are known adhesion molecules. Many previous reports have clearly demonstrated that reduced expression of these molecules is associated with benign and/or malignant cell proliferation, cell migration, tumor invasion, cancer metastasis, and chemotherapy resistance in a wide array of cells and tissues [11, 12, 14]. Previous studies have shown that alteration of adhesion molecule expressions in endometrial tissues may play a role in the etiopathogenesis of benign and malignant conditions such as embryo implantation, spontaneous abortion, and endometrial cancer progression. Leblanc et al. reported that E-cadherin and N-cadherin expressions were significantly lower and CD44v3 expression was significantly higher in the endometrial tissue samples of women with endometrial cancer than in those of controls [30]. Additionally, Poncelet et al. reported that infertile women with hydrosalpinges had decreased expressions of E-cadherin and N-cadherin in their endometrial tissue samples in comparison with fertile control subjects [31], whereas CD44 expression was similar between these groups. Sahin et al. demonstrated that placental tissue expression of P-cadherin was lower in the trophoblast cells of women who had experienced spontaneous abortions than in those of ectopic pregnancy and control subjects [32].

On the other hand, only a few studies have examined adhesion molecule expression in the eutopic and ectopic endometrial tissues of women with endometriosis, and the studies that have examined this subject yielded controversial results. Some of these studies demonstrated that both CD44 and E-cadherin expressions were higher in the eutopic and/or ectopic endometrial tissues of women with endometriosis than in control endometrial tissues [33–35]. Contrary to these reports, Poncelet et al. found that the expression of CD44 and E-cadherin in epithelial cells was lower in cases of peritoneal endometriosis than in normal endometrium [36]. In the present study, we found that both CD44 and E-cadherin expressions were significantly lower in ectopic endometrial tissues than in control endometrial tissues. These conflicting results between studies may have been due to differences in the demographic features of the studied populations or in the investigated subtypes of endometriosis. To reduce the probability of error, we conducted the study in a homogeneous group with a single subtype of endometriosis. Moreover, we also found that CD44 expression was negatively correlated with the presence of peritoneal endometriotic lesions, whileE-cadherine expression was negatively correlated with the presence of deep infiltrating endometriosis. These correlations indicated that different expressions of adhesion molecules may contribute to the generation of the different subtype of the endometriosis.

Nuclear factor-kappa B (NF-kB) has been shown to critically increases the ability of endometriotic cells to invade and adhere to the peritoneal surface by early regulation of the inflammatory response in endometriotic tissues [37]. Activation of the NF-kB pathway mediates cell proliferation and angiogenesis. Moreover, previous cancer cell line studies have demonstrated that NF-kB stimulates the self-renewal mechanisms of cancer stem cells, which helping to maintain c*ancer stem sell* (CSC)’ populations in tumors [38]. Similarly, a recent study clearly demonstrated that inhibition of NF-kB reduces the expression of stem cell transcription factors SOX-2, NANOG, and Oct-4 in CSCs that present overexpression in several cancers, including breast, prostate, and oral squamous cell carcinoma [39]. NF-kB activation has also been associated with the invasive and metastatic capabilities of cancer, mainly by modulating the epithelial to mesenchymal transition, which reduces the epithelial marker E-cadherin [40]. The overexpression of endometrial stem cell marker Oct-4, the decreased expression of adhesion molecules (especially E-Cadherin), and the correlation of these expressions with the clinicopathological findings of he present study demonstrated that these molecular markers may contribute to the generation and progression of endometriosis.

Regarding the cyclic fluctuation of CD44 and E-cadherin expressions in endometrial tissue, previous studies have shown that these expressions were lowest during the proliferative phase of menstruation and highest during the late secretory phase of menstruation [34, 36]. Similarly, our cyclic fashion analysis demonstrated that the expression of CD44 and E-cadherin in the endometrial tissue tended to be lower in the proliferation phase of the menstrual cycle and higher in the secretory phase. We also found that during the secretory phase of the menstrual cycle, the expression of adhesion molecules was lower in the eutopic endometrial tissues of women with endometriosis than in the control group. However, there were no significant differences between the different phases of menstruation and the expressions of these adhesion molecules in the eutopic and control endometrial tissues. Previous studies have indicated that these adhesion molecules might be involved in the implantation of fertilized ovum in the endometrium [41]. In light of these studies, decreased expression of adhesion molecules in the secretory phase of the menstrual cycle may contribute to the development of infertility in women with endometriosis.

The main limitation of the present study was the relatively small number of participants. However, this is the first study to investigate the stem cells and adhesion molecules expressions in the eutopic and ectopic endometrial tissue samples of patients with a single subtype of endometriosis.

## Conclusions

The combination of an increased endometrial stem cell population and altered adhesion molecule expression in endometriotic tissue appears to contribute to the development and progression of endometriosis. To fully investigate the etiology of and therapeutic approaches to endometriosis, further animal model studies are recommended to test the inhibition of endometrial stem cells self-renewal and related pathways associated with adhesion molecule expression.

## Data Availability

The datasets used and/or analysed during the current study are available from the corresponding author on reasonable request.

## Abbreviations

Oct-4: Octamer-binding protein 4
CD44: A cell-surface glycoprotein
ASRM: American Society for Reproductive Medicine
IHC: Immunohistochemisty
SOX-2: Sex determining region Y-box 2
c-KIT: Tyrosine-protein kinase KIT, CD117
NF-kB: Nuclear factor-kappa B
CSC: *Cancer Stem Cell*
NANOG: A transcription factor in embryonic stem cells
SD: Standard deviation

## References

[1] Othman ER, Hornung D, Hussein M, Abdelaal II, Sayed AA, Fetih AN (2016). Soluble tumor necrosis factor-alpha receptors in the serum of endometriosis patients. Eur J Obstet Gynecol Reprod Biol.

[2] Boiani M, Eckardt S, Schöler HR, McLaughlin KJ (2002). Oct4 distribution and level in mouse clones: consequences for pluripotency. Genes Dev.

[3] Nichols J, Zevnik B, Anastassiadis K, Niwa H, Klewe-Nebenius D, Chambers I (1998). Formation of pluripotent stem cells in the mammalian embryo depends on the POU transcription factor Oct4. Cell..

[4] Niwa H, Miyazaki J, Smith AG (2000). Quantitative expression of Oct-3/4 defines differentiation, dedifferentiation or self-renewal of ES cells. Nat Genet.

[5] Chang J-H, Au H-K, Lee W-C, Chi C-C, Ling T-Y, Wang L-M (2013). Expression of the pluripotent transcription factor OCT4 promotes cell migration in endometriosis. Fertil Steril.

[6] Tempest N, Maclean A, Hapangama DK. Endometrial stem cell markers: current concepts and unresolved questions. Int J Mol Sci. 2018;19(10):3240. 10.3390/ijms19103240. 10.3390/ijms19103240 PMC6214006 30347708

[7] Tai M-H, Chang C-C, Kiupel M, Webster JD, Olson LK, Trosko JE (2005). Oct4 expression in adult human stem cells: evidence in support of the stem cell theory of carcinogenesis. Carcinogenesis..

[8] Bentz E-K, Kenning M, Schneeberger C, Kolbus A, Huber JC, Hefler LA (2010). OCT-4 expression in follicular and luteal phase endometrium: a pilot study. Reprod Biol Endocrinol RBE.

[9] Siufi Neto J, Kho RM, dos Santos Siufi DF, Baracat EC, Anderson KS, Abrão MS (2014). Cellular, histologic, and molecular changes associated with endometriosis and ovarian cancer. J Minim Invasive Gynecol.

[10] Gazvani R, Templeton A (2002). New considerations for the pathogenesis of endometriosis. Int J Gynaecol Obstet Off Organ Int Fed Gynaecol Obstet.

[11] Okamoto I, Kawano Y, Tsuiki H, Sasaki J, Nakao M, Matsumoto M (1999). CD44 cleavage induced by a membrane-associated metalloprotease plays a critical role in tumor cell migration. Oncogene..

[12] Nothnick WB, Fan F, Iczkowski KA, Ashwell R, Thomas P, Tawfik OW (2001). CD44s expression is reduced in endometriotic lesions compared to eutopic endometrium in women with endometriosis. Int J Gynecol Pathol Off J Int Soc Gynecol Pathol.

[13] Günthert U, Stauder R, Mayer B, Terpe HJ, Finke L, Friedrichs K (1995). Are CD44 variant isoforms involved in human tumour progression?. Cancer Surv.

[14] Naor D, Sionov RV, Ish-Shalom D (1997). CD44: structure, function, and association with the malignant process. Adv Cancer Res.

[15] van der Linden PJ, de Goeij AF, Dunselman GA, van der Linden EP, Ramaekers FC, Evers JL (1994). Expression of integrins and E-cadherin in cells from menstrual effluent, endometrium, peritoneal fluid, peritoneum, and endometriosis. Fertil Steril.

[16] Takeichi M (1991). Cadherin cell adhesion receptors as a morphogenetic regulator. Science..

[17] Wheelock MJ, Shintani Y, Maeda M, Fukumoto Y, Johnson KR (2008). Cadherin switching. J Cell Sci.

[18] Risinger JI, Berchuck A, Kohler MF, Boyd J (1994). Mutations of the E-cadherin gene in human gynecologic cancers. Nat Genet.

[19] Usta A, Turan G, Adali E (2017). The expression of Cyclophilin a in ovarian Endometrioma: its correlation with recurrence and vascularity. Tohoku J Exp Med.

[20] Murray MJ, Meyer WR, Zaino RJ, Lessey BA, Novotny DB, Ireland K (2004). A critical analysis of the accuracy, reproducibility, and clinical utility of histologic endometrial dating in fertile women. Fertil Steril.

[21] Figueira PGM, Abrão MS, Krikun G, Taylor HS, Taylor H (2011). Stem cells in endometrium and their role in the pathogenesis of endometriosis. Ann N Y Acad Sci.

[22] Sampson JA (1927). Metastatic or embolic endometriosis, due to the menstrual dissemination of endometrial tissue into the venous circulation. Am J Pathol.

[23] Gruenwald P (1942). Origin of endometriosis from the mesenchyme of the celomic walls. Am J Obstet Gynecol.

[24] Sasson IE, Taylor HS (2008). Stem cells and the pathogenesis of endometriosis. Ann N Y Acad Sci.

[25] Schöler HR, Ruppert S, Suzuki N, Chowdhury K, Gruss P (1990). New type of POU domain in germ line-specific protein Oct-4. Nature..

[26] Okita K, Ichisaka T, Yamanaka S (2007). Generation of germline-competent induced pluripotent stem cells. Nature..

[27] Sotomayor P, Godoy A, Smith GJ, Huss WJ (2009). Oct4A is expressed by a subpopulation of prostate neuroendocrine cells. Prostate.

[28] Matthai C, Horvat R, Noe M, Nagele F, Radjabi A, van Trotsenburg M (2006). Oct-4 expression in human endometrium. Mol Hum Reprod.

[29] Pacchiarotti A, Caserta D, Sbracia M, Moscarini M (2011). Expression of oct-4 and c-kit antigens in endometriosis. Fertil Steril.

[30] Leblanc M, Poncelet C, Soriano D, Walker-Combrouze F, Madelenat P, Scoazec JY (2001). Alteration of CD44 and cadherins expression: possible association with augmented aggressiveness and invasiveness of endometrial carcinoma. Virchows Arch.

[31] Poncelet C, Cornelis F, Tepper M, Sauce E, Magan N, Wolf JP (2010). Expression of E- and N-cadherin and CD44 in endometrium and hydrosalpinges from infertile women. Fertil Steril.

[32] Şahin H, Akpak YK, Berber U, Gün İ, Demirel D, Ergür AR (2014). Expression of P-cadherin (cadherin-3) and E-selectin in the villous trophoblast of first trimester human placenta. J Turk Ger Gynecol Assoc.

[33] Pazhohan A, Amidi F, Akbari-Asbagh F, Seyedrezazadeh E, Aftabi Y, Abdolalizadeh J (2018). Expression and shedding of CD44 in the endometrium of women with endometriosis and modulating effects of vitamin D: a randomized exploratory trial. J Steroid Biochem Mol Biol.

[34] Matsuzaki S, Darcha C, Maleysson E, Canis M, Mage G (2010). Impaired down-regulation of E-cadherin and beta-catenin protein expression in endometrial epithelial cells in the mid-secretory endometrium of infertile patients with endometriosis. J Clin Endocrinol Metab.

[35] Knudtson JF, McLaughlin JE, Santos MT, Binkley PA, Tekmal RR, Schenken RS (2019). The hyaluronic acid system is intact in menstrual endometrial cells in women with and without endometriosis. Reprod Sci Thousand Oaks Calif.

[36] Poncelet C, Leblanc M, Walker-Combrouze F, Soriano D, Feldmann G, Madelenat P (2002). Expression of cadherins and CD44 isoforms in human endometrium and peritoneal endometriosis. Acta Obstet Gynecol Scand.

[37] Kaponis A, Iwabe T, Taniguchi F, Ito M, Deura I, Decavalas G (2012). The role of NF-kappaB in endometriosis. Front Biosci Sch Ed.

[38] Shostak K, Chariot A (2011). NF-κB, stem cells and breast cancer: the links get stronger. Breast Cancer Res BCR.

[39] Ben-Porath I, Thomson MW, Carey VJ, Ge R, Bell GW, Regev A (2008). An embryonic stem cell-like gene expression signature in poorly differentiated aggressive human tumors. Nat Genet.

[40] Chua HL, Bhat-Nakshatri P, Clare SE, Morimiya A, Badve S, Nakshatri H (2007). NF-kappaB represses E-cadherin expression and enhances epithelial to mesenchymal transition of mammary epithelial cells: potential involvement of ZEB-1 and ZEB-2. Oncogene..

[41] Yaegashi N, Fujita N, Yajima A, Nakamura M (1995). Menstrual cycle dependent expression of CD44 in normal human endometrium. Hum Pathol.

